# COVID-19 mortality across occupations and secondary risks for elderly individuals in the household: A population register-based study

**DOI:** 10.5271/sjweh.3992

**Published:** 2021-12-30

**Authors:** Sunnee Billingsley, Maria Brandén, Siddartha Aradhya, Sven Drefahl, Gunnar Andersson, Eleonora Mussino

**Affiliations:** 1 Stockholm University Demography Unit, Department of Sociology, Stockholm University; 2 Institute for Analytical Sociology (IAS), Linköping University

**Keywords:** corona virus, COVID-19 risk group, elderly mortality risk, occupational exposure, population study, work from home

## Abstract

**Objectives:**

This is the first population-level study to examine inequalities in COVID-19 mortality according to working-age individuals’ occupations and the indirect occupational effects on COVID-19 mortality of older individuals who live with them.

**Methods:**

We used early-release data for the entire population of Sweden of all recorded COVID-19 deaths from 12 March 2020 to 23 February 2021, which we linked to administrative registers and occupational measures. Cox proportional hazard models assessed relative risks of COVID-19 mortality for the working-aged population registered in an occupation in December 2018 and the older population who lived with them.

**Results:**

Among working aged-adults, taxi/bus drivers had the highest relative risk of COVID-19 mortality: over four times that of skilled workers in IT, economics, or administration when adjusted only for basic demographic characteristics. After adjusting for socioeconomic factors (education, income and country of birth), there are no occupational groups with clearly elevated (statistically significant) COVID-19 mortality. Neither a measure of exposure within occupations nor the share that generally can work from home were related to working-aged adults’ risk of COVID-19 mortality. Instead of occupational factors, traditional socioeconomic risk factors best explained variation in COVID-19 mortality. Elderly individuals, however, faced higher COVID-19 mortality risk both when living with a delivery or postal worker or worker(s) in occupations that generally work from home less, even when their socioeconomic factors are taken into account.

**Conclusions:**

Inequalities in COVID-19 mortality of working-aged adults were mostly based on traditional risk factors and not on occupational divisions or characteristics in Sweden. However, older individuals living with those who likely cannot work from home or work in delivery or postal services were a vulnerable group.

Individuals who have been particularly vulnerable to succumbing to COVID-19 include the elderly, men, ethnic minorities, and people with low educational attainment or existing illnesses (1–3). Research also clearly points to occupational differences in how the virus is spread: In Italy and the US, studies have shown that frontline healthcare workers alone made up 10–20% of all infections (4, 5). In Sweden, bus drivers, taxi drivers and pizza makers have a significantly higher risk of infection than other workers (6). The risk of infection and death from the infection are two separate situations, however, and we know little about occupational differences in COVID-19 mortality. Some evidence points to workers in frontline or essential occupations carrying a higher risk of COVID-19 mortality, for example in California (7), Massachusetts (8), and England/Wales (9) in the early-to-mid stage of the pandemic. Given that we know traditional risk factors are not distributed equally across occupations, we can improve public health responses by assessing whether individuals’ work situation contributes to mortality differentials or if these risk factors operate independently of occupational exposure to coronavirus.

For the elderly, who are most at risk of COVID-19 mortality (10, 11), the age-composition of households appears to play an important role in diffusion (12) and fatalities (13–15), and working-age individuals seem to increase the risk of COVID-19 mortality for elderly household members compared to those who do not live with a working-age adult (15). The role of occupational exposure is therefore potentially not only important for the individual worker but also for those sharing a living space. It may be a general pattern that workers put the older people they live with at risk or rather that specific occupations drive these patterns.

Differences in exposure risks are likely to emerge based on context-specific restrictions and recommendations related to slowing the spread of COVID-19. When countries have implemented strong lockdown measures, inequality in exposure risk is likely based on whether the individual is a frontline worker or not. When some or all restrictions are lifted or never implemented, inequality in risk emerges between those who can work from home versus those who cannot, as well as those working in public spaces or near the virus and those who do not. Sweden largely diverged from the international consensus on non-pharmaceutical interventions by never formally implementing a lockdown and instead relying on widespread normative compliance with social distancing of its population from the beginning of the pandemic. Despite not mandating a lockdown, however, Google’s COVID-19 Community Mobility Reports indicate that mobility trends for workplaces decreased 25% in the country as a whole and 36% in Stockholm in March and April 2020 (16), suggesting some change of behavior in response to the global pandemic. Also, unlike other contexts, the government did not recommend personal protective equipment (PPE) such as facemasks to the public until late in the pandemic (January 2021) (17), and only then in relation to taking public transportation. The pandemic has been severe in Sweden, where COVID-19 related deaths far outnumber those in neighboring and similar Nordic contexts. Taken together, Sweden provides a unique context for assessing occupational inequalities in COVID-19 mortality.

This is the first population-level study to examine inequalities in COVID-19 mortality according to working age individuals’ occupations, characteristics of their occupation, and the indirect effect of this occupation on COVID-19 mortality of older individuals with whom they live. Using Swedish individual population registers, we additionally assess how occupations and their characteristics relate to the risk factors previously identified for COVID-19 mortality in Sweden. Policy responses may differ depending on whether the occupation itself poses specific risks or if COVID-19 mortality is grouped within occupations due to compositional factors.

## Methods

We used the Swedish administrative and population registers that include individual-level data on a wide range of socioeconomic, demographic, and residential characteristics of all individuals living in Sweden during December 2019, and who had been resident in Sweden for at least two years. This information is linked through unique identification numbers to the cause of death register updated up until 23 February 2021, which enables us to distinguish recorded COVID-19 mortality from other causes of death.

We selected two populations for our analyses: (i) all working age individuals (20–66 years at the time of the first observation, 12 March 2020), who were registered with an occupation in December 2018 (N=4 620 395); (ii) individuals aged ≥67 years (a common retirement age in Sweden) on 12 March 2020 and living in a household (in December 2019) with at least one person aged 20–66 who was registered with an occupation in December 2018 (N=209 229). See supplementary material (https://www.sjweh.fi/article/3992) figure S1 for a description of exclusion of cases.

This study is produced under the Swedish Statistics Act, where privacy concerns restrict the availability of register data for research. Aggregated data can be made available by the authors, conditional on ethical vetting. The authors access the individual-level data through Statistics Sweden’s micro-online access system MONA. The Swedish ethical-vetting authority has approved the analyses, Dnr 2020-02199.

### Outcome variable

We use data on all deaths reported between 12 March 2020 (the date of the first confirmed death by COVID-19 in Sweden) and 23 February 2021, and whether each death was associated with COVID-19. The data on deaths contain all individuals who lived in Sweden and had been a resident in Sweden for at least two years. These data were collected by the Swedish National Board of Health and Welfare, the agency responsible for the cause of death register. In the study population of working individuals and the elderly people living with them, 12 103 individuals in our analytical sample died during the study period; the Swedish National Board of Health and Welfare reported 1355 of these as COVID-19 deaths. Of these deaths, COVID-19 was identified as the underlying cause of death in 1210 cases (ICD-10 code U07.1: 1173 deaths; U07.2: 35 deaths; or B3.42: 2 deaths). Of the remaining 145 cases, ICD-10 codes U07.1, U07.2 or B3.42 were listed as contributing causes of death but not the underlying cause of death. Our data capture two full peaks and the beginning of a third in COVID-19 mortality in Sweden and therefore the great majority of deaths in our study population in Sweden.

### Occupational measures:

We applied three different measures related to occupation. Using the Swedish occupational registers, we constructed occupational groups that are widely considered to be frontline and/or essential occupations (6, 18) and in particular in the case of Sweden (6): care workers, police officers and security guards, service sector personnel, delivery workers, taxi- and bus drivers, teachers, meat packers, and cleaners. We compared the COVID-19 mortality risk of these workers (or the older individuals who live with them) to skilled workers in IT, economics, or administration, which are a large group of workers who are not considered frontline, as well as to all other occupations combined. The occupational group approach allowed us to isolate specific groups who are at risk. For a full list of the SSYK 2012 (the Swedish equivalent of ISCO-08) in each occupation, see supplementary table S1.

Whereas the frontline and/or essential worker categories focus on those who were generally required to continue working during the pandemic, our second measure focused on those who may be particularly at risk while working. It is an index combining three work context indicators, all of which are relevant to the spread of COVID-19: how much the job requires contact with others, how close the physical proximity is to people, and the frequency of exposure to disease and infection. The measure is based on publicly available data through the O*NET online database (version 24.2) (www.onetonline.org) supported by the US Department of Labor/Employment and Training Administration. O*NET data has been applied in scientific research on health outcomes (19) as well as widely discussed in reports and media in relation to COVID-19 (20–24). The occupational exposure information has been constructed for the Standard Occupational Classification System (SOC) in the US and we matched SOC codes to the International Standard Classification of Occupations (ISCO-08). We first used the crosswalk procedure provided by Hardy et al (26), and then matched ISCO-08 codes to the Swedish Standard Classification of Occupations (SSYK 2012) with the occupational code key provided by Statistics Sweden (25). The survey questions on which the measures were constructed by O*NET, as well as the specific example of how our measure was derived for taxi drivers is presented in supplementary figure S2. Answers to these questions were standardized. As we had no basis for expecting any of these three work context dimensions to be more important than the other, we generated an unweighted mean to arrive at our occupational exposure index. The index is measured on a continuous scale of 0–100, with 100 representing constant exposure to infection, contact with others and near physical proximity. The highest score (98.7) is found for dental hygienists and the lowest score (23.8) is found for debt collectors. Supplementary figure S3 shows the distribution of our study populations across the occupational exposure measure as the share of the total, with noted examples of specific occupations.

In addition to the occupational groups and the continuous measure capturing exposure, we use a measure intended to capture the possibility of working at home in a given occupation. The measure is derived from the European Labor Force Survey (EULFS) for Sweden 2018, and constructed by the percentage in a given occupation (3 digit ISCO-08 merged to 3 digit SSYK 12) who responded that they never work from home. This measure reflects the share within an occupation in usual times and is not specific to the coronavirus pandemic, which likely means that it is a lower estimate of the share that actually were able to work from home when work was restructured due to the pandemic. Nevertheless, it should measure the overall capacity of an occupation to shift away from the workplace in times of need.

For the population aged 20–66 years, we measured one’s own primary occupation, whereas for the population aged ≥67 years, we measured the primary occupation of other individuals aged 20–66 in the household. If there were more than one individual with an occupation in an elderly individual’s household, we let any frontline/essential occupation dominate.

In the baseline models, we controlled for age, sex and whether the individual was living in Stockholm (measured at the end of 2019). In fully adjusted models, we additionally controlled for potential confounders and mediators: country of birth, highest achieved educational degree, and individual net income (measured at the end of 2018).

We performed Cox proportional hazard regressions with COVID-19 death as an event, with the log of age as an offset in the models (27). The follow-up time began 12 March 2020 and ended with (i) all-cause mortality between starting time and 23 February 2021 (the last reliable COVID-19 death in our data was reported at this date), or (ii) being alive on 24 February 2021. All analyses were conducted using Stata Statistical Software: Release 16 (StataCorp LP, College Station, TX, USA).

## Results

Descriptive statistics of the population and covariates are available in supplementary table S2. Full model results for the working-aged and older populations are available in supplementary tables S3 and S4, respectively. The first figures display both the relationship between COVID-19 mortality and our three occupational measures, assessed independently, and how occupational differences in mortality are mediated or confounded by our set of socioeconomic status (SES) control variables (educational attainment, income and country of birth). We interpreted results both in terms of 95% and 90% confidence intervals (CI) because of the few numbers of deaths distributed over the various occupational categories. In all tables, however, only results according to 95% CI are reported.

**Figure 1:**
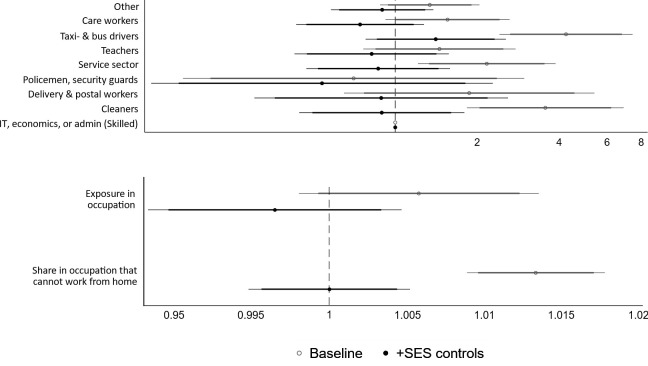
Relative risks from Cox proportional hazard models, occupational differences with and without adjusting for mediators and confounders, aged 20–66 years with a registered occupation. Note: meat packers are not shown in this figure as there were no COVID-19 deaths reported in this category. Exposure in occupation is the O*NET measure. Both measures in the lower panel (exposure and share in occupations that cannot work from home) are continuous measures ranging from 0–100.

For working-aged people, figure 1 shows that, without adjusting for SES (light grey lines), the occupations that are typically considered to be frontline are in general at a higher risk of COVID-19 mortality than skilled workers in IT, economics or administration. The only exception was the occupation of police/guard, for whom the estimated risk of COVID-19 mortality was lower than skilled workers. Taxi/bus drivers, service sector workers and cleaners have the highest relative risks of COVID-19 mortality. Taxi/bus drivers have over four times that of the skilled workers group. Net of SES (dark grey lines), taxi/bus drivers’ mortality risk remains the highest [relative risk (RR) 1.41], whereas all other occupational groups shift to having a lower risk of COVID-19 mortality. The increased mortality risk for taxi/bus drivers is no longer statistically significant (P=0.26).

Occupational exposure (lower panel of figure 1), measuring closeness to/contact with others and proximity to infectious diseases, relates positively to COVID-19 mortality risk, but this estimate is not statistically significant. When adjusting for SES, the relationship shifts to below 1 (indicating a lower risk of COVID-19 mortality). As a robustness check, we relaxed the assumption of linearity and estimated the relationship with a quadratic and cubic term instead. These transformations did not change the result.

The share of individuals who cannot work from home in their occupation is more positively related to COVID-19 mortality than exposure in the occupation. This relationship also disappears when adjusting for SES.

**Figure 2:**
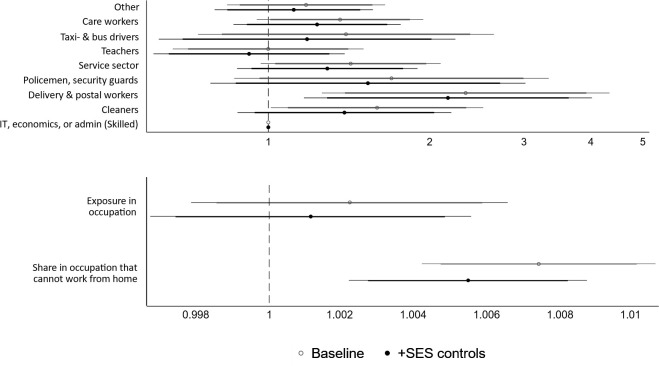
Relative risks from Cox proportional hazard models, occupational differences with and without adjusting for mediators and confounders, ages 67+ living with a person <67 with a registered occupation. Note: meat packers are not shown in this figure as there were no COVID-19 deaths reported in this category. Exposure in occupation is the O*NET measure. Both measures in the lower panel (exposure and share in occupations that cannot work from home) are continuous measures ranging from 0–100.

For older individuals who live with working-aged adult(s), figure 2 shows a few different patterns related to occupational differences. First, living with a taxi/bus driver does not add additional risk of COVID-19 mortality. Living with a cleaner or delivery and postal worker does increase the risk of COVID-19 mortality for older people, and if we consider differences using a 10% significance level, service sector and care workers are also associated with higher older age COVID-19 mortality. When adjusting for SES, the only occupational group that posed a higher risk of COVID-19 mortality for older co-residents was delivery and postal workers (adjusted for SES: RR 2.16, P=0.015). Occupational exposure was positively related to mortality risk, but this relationship was not statistically significant. When considering the importance of working from home, we see that elderly individuals who were living with worker(s) who likely cannot work from home have a higher risk of COVID-19 mortality. This relationship persisted when we adjust for the older person’s own SES (RR 1.005, P=0.001). This RR is for an increase in being able to work from home of only one percentage point, whereas the RR is 1.73 if we consider instead 100% versus 0% of an occupation not being able to work from home.

Tables 1 and 2 show how the relationship between COVID-19 and education, income and country of birth changes with and without adjusting for occupational information. Worth noting is that adding occupational information to the baseline model does not improve the model fit for either Akaike’s or Bayesian information criteria (AIC/BIC), which implies that traditional risk factors such as SES explain variation in COVID-19 mortality better than occupational factors and that occupational information does not contribute much to understanding COVID-19 mortality beyond what we learn from SES. This finding was consistent across both the working-aged population and the older individuals who live with a working-aged individual. We can conclude then that much, if not all, of the relationship between COVID-19 mortality and occupations or their characteristics is compositional. The only exception was a lower AIC and similar BIC in the models with older individuals when adding the share that can work from home.

**Table 1:** Relative risks (RR) from Cox proportional hazard models, socioeconomic indicators with and without adjusting for occupational information, aged 20–66 years with a registered occupation (N=4 620 395; N with COVID deaths=409). [SE=standard error; HIC=high income countries; LMIC=low and middle income countries; MENA=Middle East and North Africa; AIC=Akaike’s information criteria; BIC=Bayesian information criterial.]

	Model 1			Model 2			Model 3			Model 4		
			
	Baseline model (AIC=8919; BIC=9052)			Adjusted for occupational groups (AIC=8927; BIC=9167)			Adjusted for exposure in occupation (AIC=8919; BIC=9066)			Adjusted for work from home (AIC=8920; BIC=9067)		
			
	RR	SE	P-value	RR	SE	P-value	RR	SE	P-value	RR	SE	P-value
Education												
Primary	1.09	0.17	0.567	1.09	0.17	0.573	1.08	0.17	0.637	1.09	0.18	0.585
Secondary	1.13	0.13	0.277	1.13	0.13	0.302	1.13	0.13	0.300	1.13	0.14	0.307
Post-secondary	1			1			1			1		
Missing	0.54	0.39	0.391	0.55	0.40	0.407	0.53	0.38	0.379	0.54	0.39	0.391
Country of birth												
Sweden	1			1			1			1		
HIC	1.49	0.28	0.033	1.50	0.28	0.033	1.50	0.28	0.032	1.49	0.28	0.034
LMIC other	3.91	0.50	0.000	3.86	0.51	0.000	3.94	0.51	0.000	3.90	0.52	0.000
LMIC MENA	3.20	0.55	0.000	3.10	0.55	0.000	3.26	0.56	0.000	3.20	0.56	0.000
Income												
Lowest tertile	2.51	0.38	0.000	2.52	0.39	0.000	2.53	0.38	0.000	2.51	0.39	0.000
Mid tertile	2.07	0.24	0.000	2.07	0.25	0.000	2.10	0.25	0.000	2.07	0.25	0.000
Highest tertile	1			1			1			1		

**Table 2:** Relative risks (RR) from Cox proportional hazard models, socioeconomic indicators with and without adjusting for occupational information, aged ≥67 years living with a person <67 years with a registered occupation. (N=209 229; N with COVID deaths=946). [SE=standard error; HIC=high income countries; LMIC=low and middle income countries; MENA=Middle East and North Africa; AIC=Akaike’s information criteria; BIC=Bayesian information criterial.]

	Model 1			Model 2			Model 3			Model 4		
			
	Baseline model (AIC=14 831; BIC=14 934)			Adjusted for occupational groups (AIC=14 833; BIC=15 018)			Adjusted for exposure in occupation (AIC=14 833; BIC=14 946)			Adjusted for work from home (AIC=14 822; BIC=14 935)		
			
	RR	SE	P-value	RR	SE	P-value	RR	SE	P-value	RR	SE	P-value
Education												
Primary	1.34	0.13	0.003	1.32	0.13	0.005	1.34	0.13	0.003	1.28	0.13	0.014
Secondary	1.34	0.13	0.002	1.33	0.13	0.003	1.34	0.13	0.002	1.30	0.13	0.006
Post-secondary	1			1			1			1		
Missing	0.90	0.15	0.501	0.88	0.14	0.413	0.89	0.15	0.487	0.86	0.14	0.359
Country of birth												
Sweden	1			1			1			1		
HIC	1.17	0.12	0.112	1.17	0.12	0.122	1.17	0.12	0.114	1.17	0.12	0.124
LMIC other	1.69	0.19	0.000	1.66	0.19	0.000	1.68	0.19	0.000	1.64	0.19	0.000
LMIC MENA	1.93	0.28	0.000	1.92	0.28	0.000	1.92	0.28	0.000	1.88	0.28	0.000
Income												
Lowest tertile	1.28	0.15	0.034	1.27	0.15	0.041	1.28	0.15	0.034	1.25	0.15	0.060
Mid tertile	1.28	0.15	0.035	1.27	0.15	0.040	1.28	0.15	0.035	1.25	0.15	0.051
Highest tertile	1			1			1			1		

The relatively larger impact from traditional risk factors as compared to occupational characteristics is also confirmed by the results presented in tables 1 and 2. These models are similar to those in figures 1 and 2 and explore whether occupational factors mediate or confound the main risk factors that have been identified with COVID-19 mortality. The change in RR over the models adjusting for occupation or occupational characteristics is minimal. This also holds for country of origin. Results clearly show that the relationship between SES factors and COVID-19 mortality is mediated or confounded very little by occupational characteristics for those who are working in Sweden.

Table 2 shows a similar pattern for the older individuals, where the relationship between SES and COVID-19 mortality is largely robust to the addition of occupational information of the individuals with whom they live. However, when including the measure of the share who cannot work from home, the model fit slightly improved according to the AIC and there were slight reductions across most SES indicators. We can conclude from these estimates that when older people live with individuals who can work from home, they have a lower COVID-19 mortality risk, and this is independent of the SES of the older individual.

## Discussion

Our investigation into whether inequalities in COVID-19 mortality appear to be related to the work environment is motivated by the inequality in worker’s conditions and demands for showing up to work even in the midst of a pandemic. Frontline and essential workers have faced grave and uncertain consequences for their lives and families with the relentless spread of COVID-19. Our findings provide both good and bad news related to frontline workers. First, greater exposure to people and infectious disease on its own does not appear to put workers or the elderly they live with in greater danger of COVID-19 mortality. Nevertheless, we identified a few occupational groups in which COVID-19 mortality has been much higher than others. COVID-19 mortality appears to be largely clustered within occupations according to the composition of workers in terms of educational attainment, income and country of birth.

Beyond socioeconomic characteristics, one occupational group seems to be risky for the elderly who live with them: those who are ≥67 years and live with younger individuals working in delivery and postal services had an elevated risk of COVID-19 mortality. In addition, we found that working in an occupation in which the capacity to work from home is low puts older individuals in the household at a heightened risk of dying from COVID-19. Both of these heightened risks persist when adjusting for older individuals’ own SES. Even if older individuals limited their engagement with others to protect themselves during the pandemic, they may have still been vulnerable due to people continuing to work at workplaces combined with the lack of facemasks on public transportation.

Although the finding that pure exposure was not related to an elevated mortality risk may be counter­intuitive, it is plausible in light of a few factors. First, workers who are nearest to COVID-19 (doctors and nurses) are healthcare workers, who are the most likely to be provided PPE and appropriately trained in their use. These include respiratory protection, face visors, protective aprons and protective gloves. The role of PPE in protecting workers is clear: frontline healthcare workers in the US and UK had significantly higher risk of COVID-19 infection when PPE were not available or being re-used (28). Sweden adheres to the EU regulation 2016/425 on PPE and the Swedish Work Environment Agency regularly checks compliance. Although Sweden was unprepared for the increased need for PPE due to the pandemic according to a report issued by the leading medical associations and trade unions in March 2020 (29), workers with the highest occupational exposure were likely to have had some form of protection.

The finding that occupational factors for workers do not explain more variance in COVID-19 mortality than SES should be considered in light of our focus on mortality instead of infection rates. Patterns are likely to reflect the frailty and health behavior of individuals in the occupations, which correlate with socioeconomic status (30–35). We are not able to adjust for factors such as individuals being sorted into occupations on the basis of health (32, 36, 37) or experiencing health conditions directly due to their work environment (38). Worth noting, descriptive studies may overestimate the differences between occupations in COVID-19 mortality due to confounders and mediators such as education, income and country of birth.

The possibility of super-spreader events, such as professional meetings occurring early on in the pandemic, may influence estimation of occupational risks, in which the virus is transmitted in a single work environment or occupational group, such as in the case of meatpackers in Germany and miners in northern Sweden. Our extended period, including almost an entire year and three waves of COVID-19 infections, lowers that risk. We now know that bus and taxi drivers not only have a substantially heightened COVID-19 infection risk (39) but also an elevated mortality risk. The excess risk became statistically non-significant when adjusting for individual characteristics, particularly country of birth, which is likely due to low case numbers. Because taxi and bus drivers do not spend much time together and therefore are not at risk of spreading it to each other, our finding related to this occupational group is likely generalizable. Cars and buses may be hot zones for the virus as many visitors enter and exit over the course of a shift and COVID-19 does not quickly fall out of enclosed air (40). Efforts to train and provide PPE for such drivers is therefore important.

Sweden offers a good example of conditions with low government restrictions related to the spread of COVID-19. Occupational exposure likely plays a weaker role in such a context because other pathways of transmission such as restaurants, gyms, and shops remained mostly open. The extent to which our results are generalizable to other contexts may be limited as well if, for example, PPE were more widely available in Sweden or other healthcare practices were in place that protected workers better in Sweden than elsewhere. On the other hand, Sweden is also unique because it is one of the few countries that did not adopt individual mask-wearing as a practice to limit the spread of COVID-19. Were all customers to wear appropriate masks, the risk to drivers and postal workers, for example, may have been less (41). Another contextual factor to consider is whether the high-income replacement benefits for both short and long-term sick leave in Sweden influence whether individuals with poor health are in the labor market less than in contexts providing lower social benefits such as the US. This has implications for how a healthy worker effect operates within specific occupations, which would influence the differences between occupational groups, as well as how likely sickness presenteeism is, in which people who are ill do not stay home.

A few limitations of this study are important to note and involve the precision of our measures. We are not able to match occupation or income at the exact time of death. This is a problem to the extent that there was job change or a change in labor market status between the measures (December 2018) and the part of the pandemic we cover (March 2020–February 2021). We assessed the frequency of job change and labor market exit prior to the pandemic to understand how much measurement error is likely in our models. Using 2016 and 2017 as comparison, we see that 97% of working age individuals who were registered with an occupation in 2016 were also registered with one in 2017, and that 94% of these had the same occupational classifications as the one that we use in our study based on data for December 2018. Another source of measurement error relates to the measures of exposure and the share that work from home. These were both constructed in times that precede the pandemic and therefore do not capture how occupations adapted to the threat of infection. We interpret this measurement error to mean that both exposure and the share who do not work from home are generally overestimated in our data, but it is unknown how universal the overestimation is or which occupations were unable to adapt to the pandemic.

In addition, our time period covers three waves of the pandemic; no one in Sweden was vaccinated in the first and second waves, and only a small proportion of the population had been vaccinated by the end of our observation period. The results are likely not generalizable to a potential future in which vaccinations may play a more decisive role in mortality risks. To the extent that both infections and death due to infection are clustered within groups of individuals, standard errors may not be robust. Standard tools to adjust for non-independence are, however, not available given that we lack information on how observations are clustered.

In sum, our findings suggest that there are few if no real specific risk groups according to being a frontline or essential worker in a context such as Sweden in which there was no lockdown or comparably few mandated social distancing restrictions. Frontline workers may, nevertheless, still be bearing the brunt of the pandemic in Sweden even if they are not dying more. They may still be facing a higher infection risk, more sickness, extra stress, and longer work hours if more coworkers are sick.

Our findings confirm that traditional risk factors are not distributed equally across occupations. Moreover, COVID-19 mortality risk follows traditional risk factors independently of occupational factors and occupation cannot in and of itself explain observed mortality differentials among workers. However, because of our unique setting, our results cannot speak to the racial and ethnic differences emerging in other settings (42) that may be related to occupational exposure. In the US, for example, the gap between essential and non-essential workers was great in terms of who could remain at home, and this division is correlated with ethnicity and race (43). Individuals who were not born in Sweden, nevertheless, remain at higher risk of COVID-19 mortality compared to Swedish-born individuals after considering occupational factors. This is not to suggest that occupation does not contribute to the disadvantages of ethnic and racial minorities, but that inequalities are the result of more complex systemic differences (44) than can be captured by our measures. These inequalities remain an important area of future research.

## Supplementary material

Supplementary material
