# Supplementary material for: COVID-19 mortality across occupations and secondary risks for elderly individuals in the household: A population register-based study
Source: Scand J Work Environ Health. 2021 Dec 30;48(1):52–60. doi: 10.5271/sjweh.3992 (PMC8729161; doi:10.5271/sjweh.3992)

COVID-19 mortality across occupations and secondary risks for elderly individuals in the household: A population register-based study[1]

by Sunnee Billingsley, PhD,1 Maria Brandén, PhD,1, 2 Siddartha Aradhya, PhD,1 Sven Drefahl, PhD,1 Gunnar Andersson, PhD, 1 Eleonora Mussino, PhD,1

1. *Supplementary material*
2. *Correspondence to: Sunnee Billingsley, PhD, Stockholm University, Department of Sociology, Demography Unit, 106 91 Stockholm, Sweden. [E-mail: sunnee.billingsley@sociology.su.se]*

Table S1. Swedish occupational codes included in occupational groups

	**SSYK**
Care workers	221, 222, 223, 532, 533, 534; excluding 2222, 2225, 2233, 2234
Taxi- and bus drivers	8321, 8331
Meat packers	7611
Teachers	234, 5311
Service sector	522, 941, 523
Policemen, security guards	5412, 5413, 3360
Postal workers, delivery	4420, 8329
Cleaners	9111
IT, economics, or admin (skilled)	241, 242, 243, 251

Table S2. Descriptive statistics for studied population

	Aged 20-66				Aged 67+			
	N at March 12	% or mean	N dead from COVID-19	N / mean dead per 1000 from COVID-19	N at March 12	% or mean	N dead from COVID-19	N / mean dead per 1000 from COVID-19
**Exposure in occupation (mean)**	-	58.4	-	56.4	-	62.02	-	61.82
**Share never working from home in occupation (mean)**	-	0.70	-	0.73	-	0.75	-	0.78
**Occupation**								
Other	2,824,735	61.14	265	0.09	112,848	53.94	491	4.35
Care workers	601,616	13.02	37	0.06	41,107	19.65	189	4.60
Taxi- and bus drivers	38,046	0.82	25	0.66	1,899	0.91	13	6.85
Meat packers	3,409	0.07	<5	0.00	125	0.06	<5	0
Teachers	310,084	6.71	16	0.05	18,759	8.97	63	3.36
Service sector	347,283	7.52	22	0.06	15,825	7.56	91	5.75
Police men, security guards	45,004	0.97	<5	0.04	1,507	0.72	11	7.3
Postal workers, delivery	27,722	0.6	<5	0.14	1,371	0.66	14	10.21
Cleaners	86,414	1.87	14	0.16	6,292	3.01	38	6.04
IT, economics, or admin (skilled)	336,082	7.27	24	0.07	9,496	4.54	36	3.79
**Age**								
-44	2,448,299	53.0	28	0.01	-	-	-	-
45-49	548,657	11.9	29	0.05	-	-	-	-
50-54	553,912	12.0	66	0.12	-	-	-	-
55-59	494,881	10.7	88	0.18	-	-	-	-
60-64	437,273	9.5	146	0.33	-	-	-	-
65-69	137,373	3.0	52	0.38	88,545	42.3	107	1.21

70-74	-	-	-	-	66,924	32.0	157	2.35
75-79	-	-	-	-	27,640	13.2	145	5.25
80-84	-	-	-	-	13,911	6.6	174	12.51
85-89	-	-	-	-	7,868	3.8	185	23.51
90-94	-	-	-	-	3,428	1.6	130	37.92
95+	-	-	-	-	913	0.4	48	52.57
**Sex**								
Man	2,326,120	50.3	314	0.13	140,636	67.2	571	4.06
Woman	2,294,275	49.7	95	0.04	68,593	32.8	375	5.47
**Country of birth**								
Sweden	3,753,871	81.2	241	0.06	168,424	80.5	635	3.77
HIC	252,662	5.5	33	0.13	20,154	9.6	130	6.45
LMIC other	421,578	9.1	92	0.22	13,974	6.7	118	8.44
LMIC MENA	192,284	4.2	43	0.22	6,677	3.2	63	9.44
**Education**								
Primary	402,201	8.7	68	0.17	57,598	27.5	359	6.23
Secondary	2,155,991	46.7	203	0.09	84,421	40.3	342	4.05
Post-Secondary	2,025,760	43.8	136	0.07	62,120	29.7	176	2.83
Missing	36,443	0.8	<5	0.05	5,090	2.4	69	13.56
**Income**								
Lowest tertile	698,935	15.1	72	0.10	83,659	40.0	567	6.78
Mid tertile	1,789,979	38.7	188	0.11	68,204	32.6	265	3.89
Highest tertile	2,131,481	46.1	149	0.07	57,366	27.4	114	1.99
**Stockholm vs. rest**								
Rest of Sweden	3,522,148	76.2	241	0.07	164,099	78.4	555	3.38
Stockholm county	1,098,247	23.8	168	0.15	45,130	21.6	391	8.66
**TOTAL**	**4,620,395**	**100**	**409**	**0.09**	**209,229**	**100**	**946**	**4.52**

Note: Occupational characteristics refer to own occupation for individuals aged 20-66, and for household members aged 20-66 for individuals aged 67+. Occupational characteristics for household members for individuals aged 67+ are not mutually exclusive. For instance, an individual can have both a manual and a skilled worker in their household.

Table S3. Full Cox proportional hazard model of death risks, ages 20-66 with a registered occupation

	Model 1 Baseline model			Model 2 Occupational Groups			Model 3 Exposure in occupation			Model 4 Work from home		
	RR	se	p	RR	se	p	RR	se	p	RR	se	p
**Sex**												
Man	3.80	0.45	0.000	3.56	0.46	0.000	3.68	0.46	0.000	3.80	0.45	0.000
Woman	1			1			1			1		
**Residence**												
Rest of Sweden	1			1			1			1		
Stockholm	2.35	0.24	0.000	2.31	0.24	0.000	2.35	0.25	0.000	2.35	0.25	0.000
**Education**												
Primary	1.09	0.17	0.567	1.09	0.17	0.573	1.08	0.17	0.637	1.09	0.18	0.585
Secondary	1.13	0.13	0.277	1.13	0.13	0.302	1.13	0.13	0.300	1.13	0.14	0.307
Post-secondary	1			1			1			1		
Missing	0.54	0.39	0.391	0.55	0.40	0.407	0.53	0.38	0.379	0.54	0.39	0.391
**Country of birth**												
Sweden	1			1			1			1		
HIC	1.49	0.28	0.033	1.50	0.28	0.033	1.50	0.28	0.032	1.49	0.28	0.034
LMIC other	3.91	0.50	0.000	3.86	0.51	0.000	3.94	0.51	0.000	3.90	0.52	0.000
LMIC MENA	3.20	0.55	0.000	3.10	0.55	0.000	3.26	0.56	0.000	3.20	0.56	0.000
**Income**												
Lowest tertile	2.51	0.38	0.000	2.52	0.39	0.000	2.53	0.38	0.000	2.51	0.39	0.000
Mid tertile	2.07	0.24	0.000	2.07	0.25	0.000	2.10	0.25	0.000	2.07	0.25	0.000
Highest tertile	1			1			1			1		
**Occupational groups**												
Other				0.90	0.20	0.617						
Care				0.74	0.21	0.281						
Taxi/bus				1.41	0.43	0.258						
Meat packer				0.00	.	.						
Teacher				0.82	0.27	0.550						
Service				0.87	0.27	0.642						

Police, guard				0.54	0.40	0.401						
Delivery & postal				0.89	0.49	0.828						
Cleaner				0.89	0.32	0.749						
IT, economics, or admin (skilled)				1								
**Exposure in occupation**							1.00	0.00	0.394			
**Percent cannot work from home in occupation**										1.00	0.00	0.993
N covid deaths	409			409			409			409		
N	4620395			4620395			4620395			4620395		
aic	8918			8927			8919			8920		
bic	9052			9167			9066			9067		

Table S4. Full Cox proportional hazard model of death risks, ages 67+ living with a person <67 with a registered occupation

	Model 1 Baseline model			Model 2 Occupational Groups			Model 3 Exposure in occupation			Model 4 Work from home		
	RR	se	p	RR	se	p	RR	se	p	RR	se	p
**Sex**												
Man	1.61	0.12	0.000	1.61	0.12	0.000	1.61	0.12	0.000	1.61	0.12	0.000
Woman	1			1			1			1		
**Residence**												
Rest of Sweden	1			1			1			1		
Stockholm	2.35	0.16	0.000	2.35	0.17	0.000	2.35	0.17	0.000	2.39	0.17	0.000
**Education**												
Primary	1.34	0.13	0.003	1.32	0.13	0.005	1.34	0.13	0.003	1.28	0.13	0.014
Secondary	1.34	0.13	0.002	1.33	0.13	0.003	1.34	0.13	0.002	1.30	0.13	0.006
Post-secondary	1			1			1			1		
Missing	0.90	0.15	0.501	0.88	0.14	0.413	0.89	0.15	0.487	0.86	0.14	0.359
**Country of birth**												
Sweden	1			1			1			1		
HIC	1.17	0.12	0.112	1.17	0.12	0.122	1.17	0.12	0.114	1.17	0.12	0.124
LMIC other	1.69	0.19	0.000	1.66	0.19	0.000	1.68	0.19	0.000	1.64	0.19	0.000
LMIC MENA	1.93	0.28	0.000	1.92	0.28	0.000	1.92	0.28	0.000	1.88	0.28	0.000
**Income**												
Lowest tertile	1.28	0.15	0.034	1.27	0.15	0.041	1.28	0.15	0.034	1.25	0.15	0.060
Mid tertile	1.28	0.15	0.035	1.27	0.15	0.040	1.28	0.15	0.035	1.25	0.15	0.051
Highest tertile	1			1			1			1		
**Occupational groups**												
Other				1.12	0.19	0.529						
Care				1.23	0.23	0.253						
Taxi/bus				1.18	0.38	0.609						
Meat packer				0.00	.	.						
Teacher				0.92	0.19	0.694						
Service				1.29	0.26	0.200						
Police, guard				1.53	0.53	0.215						

Delivery & postal		2.16	0.68	0.015						
Cleaner		1.39	0.33	0.163						
IT, economics, or admin (skilled)		1								
**Exposure in occupation**					1.00	0.00	0.612			
**Percent cannot work from home in occupation**								1.01	0.00	0.001
N covid deaths	946	946			946			946		
N	209229	209229			209229			209229		
aic	14831	14833			14833			14822		
bic	14934	15018			14946			14935		

Figure S1. Selection flow and final population

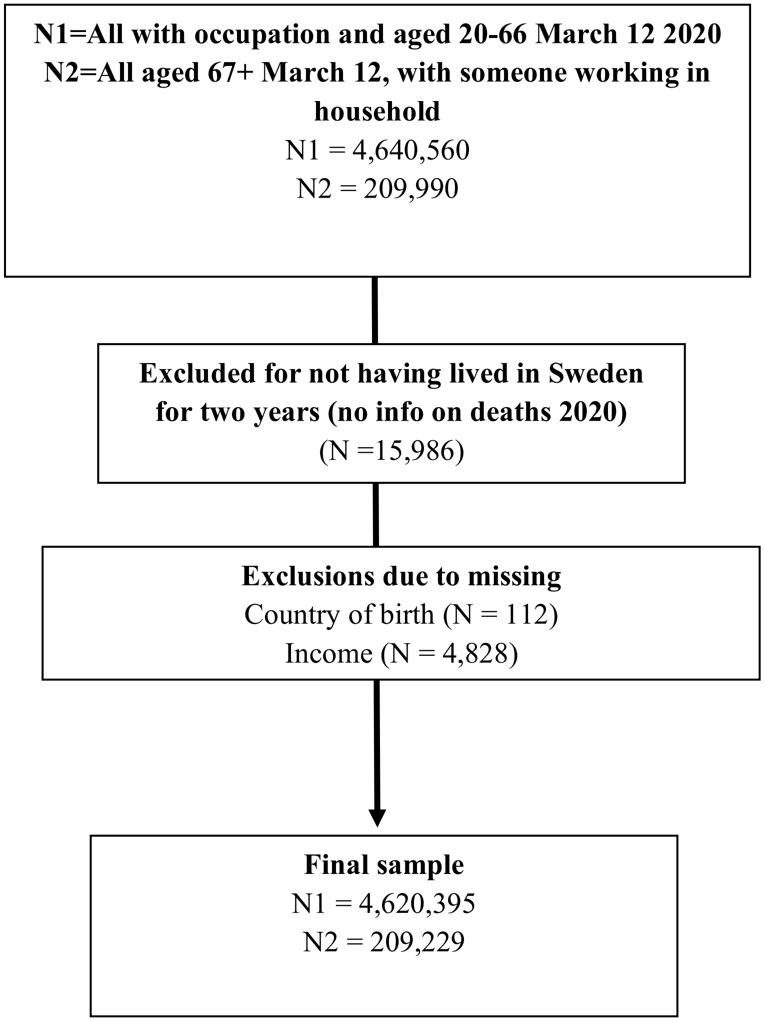

Figure S2. Occupational exposure measure origins and example

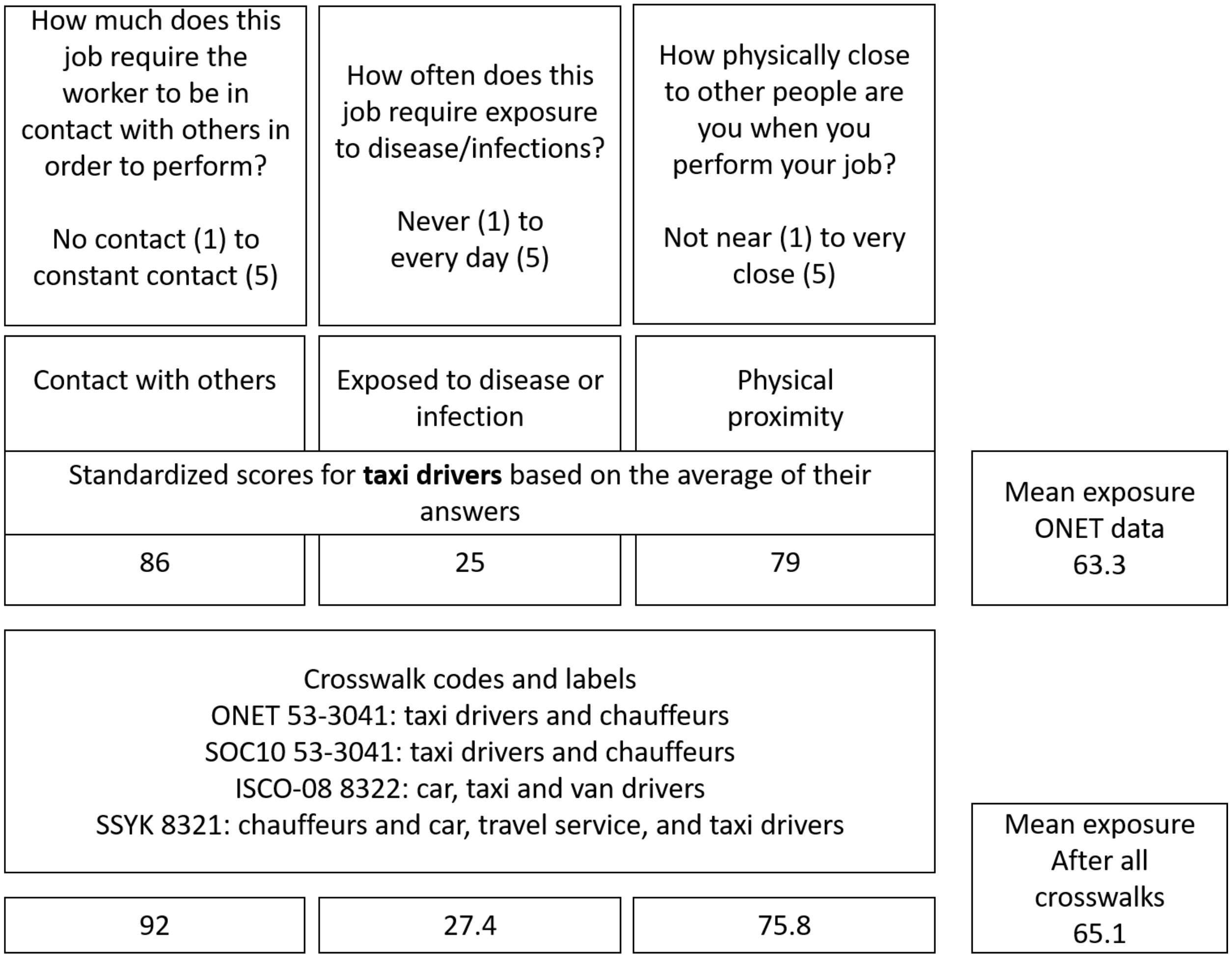

Note: The small difference in the final mean score we arrived at in comparison to the original O*NET data for taxi drivers is due to another group of drivers (light truck and delivery services) that joined taxi drivers in the crosswalk from the classification used in the US to the international classification

Figure S3. Share of study population working at various levels of occupational exposure (O*NET measure)

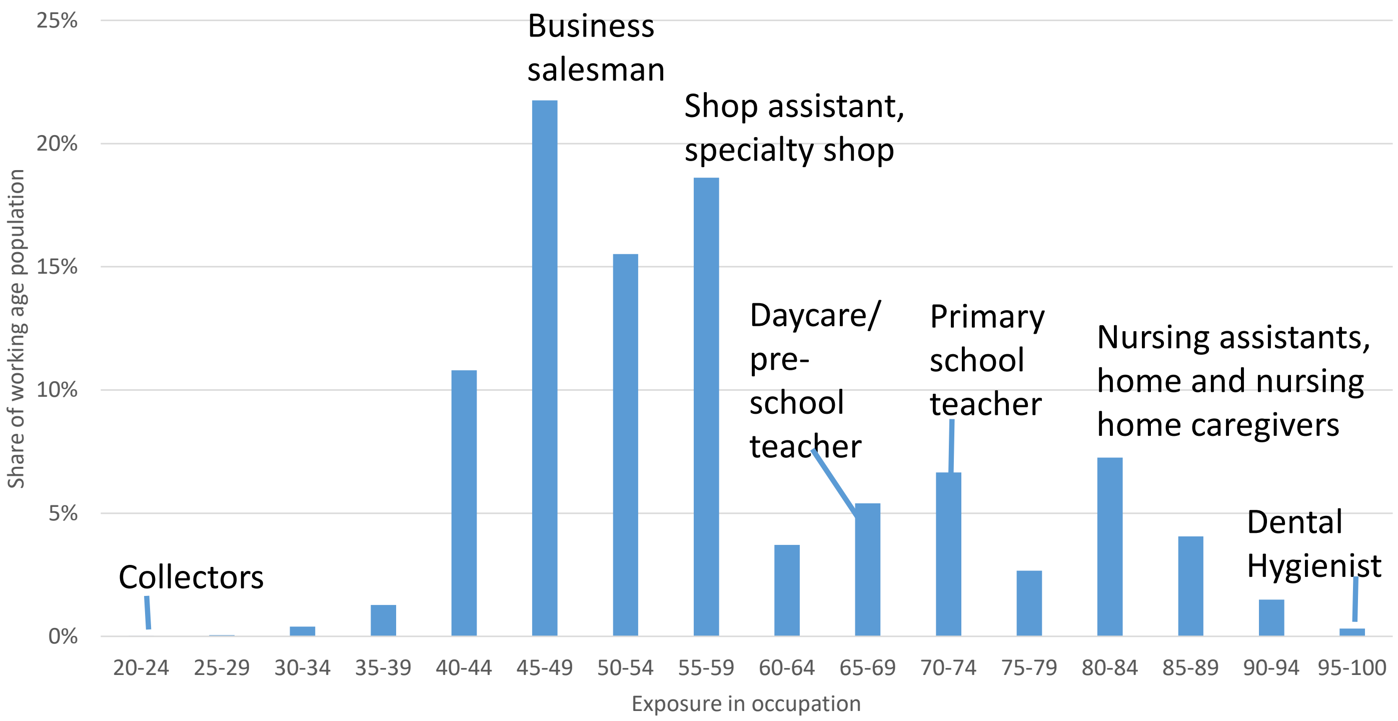

Supplement: Supplementary material [file SJWEH-48-52-S001.pdf]
